# Loss of ARID1A expression predicts poor survival prognosis in gastric cancer: a systematic meta-analysis from 14 studies

**DOI:** 10.1038/srep28919

**Published:** 2016-06-29

**Authors:** Lin Yang, Sheng Wei, Rongxian Zhao, Yingxing Wu, Hong Qiu, Huihua Xiong

**Affiliations:** 1 Department of Oncology, Tongji Hospital, Tongji Medical College, Huazhong University of Science and Technology, Wuhan, Hubei 430030, PR China; 2 Department of Epidemiology and Biostatistics, and the Ministry of Education Key Lab of Environment and Health, School of Public Health, Tongji Medical College, Huazhong University of Science and Technology, Wuhan, Hubei 430030, PR China; 3 Carilion Clinic, 1906 Belleview Ave SE, Roanoke, Virginia 24014, USA

## Abstract

The chromatin remodeling gene, AT-rich interactive domain 1A gene (*ARID1A*), frequently mutates inactively in gastric cancer (GC). However, its prognostic value remains controversial. To address this issue, a comprehensive meta-analysis was performed. Studies published until March 2016 were systematically searched. A total of 15 cohorts from 14 literatures involving 3183 patients were subjected to this meta-analysis. The pooled data showed that ARID1A expression loss predicted poor overall survival (OS) in GC (Hazard Ratio (HR) = 1.60; 95% Confidence Interval (CI) = 1.40–1.81; *P* < 0.001), with low heterogeneity among these studies (*I*^*2*^ = 21.5%; *P* = 0.214). Stratification analyses revealed that ARID1A expression loss was associated with poor OS in Asians (HR = 1.65, 95% CI = 1.44–1.89), proportion of proximal disease ≤30% subgroup (HR = 1.80, 95% CI = 1.36–2.38) and Epstein**-**Barr virus (EBV) (+) > 5% subgroup (HR = 1.59, 95% CI = 1.18–2.15). The robust results were suggested by sensitivity analyses and no evidence of significant publication bias was detected. This study demonstrated a significant relationship between deletion of ARID1A expression and poor OS in GC. Moreover, ethnicity, tumor location and EBV infection status might be potential key factors influencing this correlation.

Though the incidence has substantially declined in recent years, gastric cancer (GC) remains the third most common cause of cancer-related death globally^1^. In 2013, approximately 841,000 new cases were estimated to die from GC, among which 77% occurred in developing countries^2^. Despite great advances in early detection and comprehensive cancer treatment approaches, the overall 5-year survival rate for GC patients worldwide is still less than 25%^3^. Therefore, it is crucial to identify poor prognostic indicators for GC and guide treatment for patients with dismal outcome. Many studies have displayed that some clinical parameters, such as TNM staging, histological grade, serum tumor markers and therapeutic modalities, are prognostic factors for GC^4^,^5^,^6^,^7^. However, biological behavior of tumor cannot be sufficiently reflected by the above clinical parameters. Thus, extensive exploration of new prognostic biomarker is in progress.

AT-rich interactive domain 1A gene (*ARID1A*), encoding a large nuclear protein BAF250a, is one of vital components of the Switch/Sucrose Non Fermentable (SWI/SNF) chromatin remodeling complex^8^, which participates several nuclear activities including transcription, DNA synthesis and DNA damage repair^9^,^10^,^11^. It has been described that the great majority of ARID1A mutations in a broad spectrum of cancer lead to loss of ARID1A expression^12^,^13^,^14^,^15^,^16^, which suggests that ARID1A might behave as a tumor suppressor^12^,^17^. Moreover, functional studies present the evidence that ARID1A participates in several canonical tumor suppression processes, such as proliferation and apoptosis^12^.

Recently, next-generation sequencing (NGS) studies have revealed that the incidence of *ARID1A* mutation in patients with GC varied from 8% to 27%^18^,^19^,^20^. Meanwhile, increasing interest has been focused on determining whether *ARID1A* inactivity links to the prognosis in patients with GC. In 2011, Wang *et al*. reported that *ARID1A* mutation was independently related to better outcome in GC^18^. Later, Ibarrola-Villava M. *et al*. reported similar results^21^. Whereas other researchers suggested that ARID1A expression loss was significantly associated with shorter survival^22^,^23^, or had no prognostic effect on GC^24^. Therefore, the prognostic role of ARD1A deficiency in patients with GC remains controversial.

To date, a systematic meta-analysis, exploring the prognostic role of ARID1A expression loss in cancer including gastrointestinal cancer, has been reported^25^, but the language of literature articles analyzed in the meta-analysis was limited to English. Since half of the total GC cases occur in Eastern Asia (mainly in China) and the morbidity of GC in Asia is much higher than that in other areas^1^, it is valuable to include Chinese language articles in a meta-analysis regarding the prognosis of GC. In the present study, we sought to gain a better insight into the prognostic value of ARID1A expression loss in patients with GC through meta-analysis using literature articles published in both English and Chinese languages (from Chinese National Knowledge Infrastructure (CNKI) and Wanfang database).

## Results

### Characteristics of eligible studies

**Figure 1:**
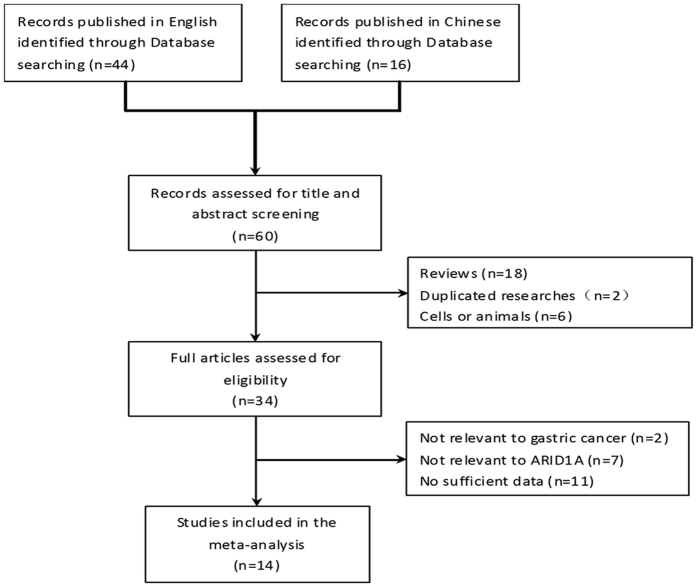
Flow chart of studies selection procedure.

Figure 1 is a flowchart showing the detailed study election procedure. A total of 44 potentially relevant publications in English and 16 publications in Chinese were initially identified by keywords search. Among these, 26 articles were excluded by two independent reviewers through title and abstract screening and 20 articles were further excluded through full articles screening. Finally, 14 publications fulfilled the criteria and were included in this meta-analysis.

The characteristics of 14 articles (15 cohorts) are summarized in Table 1. A total of fourteen studies involving 3183 patients were conducted from 2012 to March 2016. Among these, two studies (319 cases) were performed in non-Asians^21^,^26^, six studies (892 cases) in Chinese^22^,^23^,^27^,^28^,^29^,^30^, four studies (1233 cases) in Korean^24^,^31^,^32^,^33^ and two studies (739 cases) in Japanese^34^,^35^. The information on Microsatellite instability (MSI) was collected in eight studies (nine cohorts). Among them, MSI status was reported for six cohorts by immunohistochemistry of mismatch repair proteins and two cohorts by polymerase chain reaction (PCR) and electrophoresis of DNA. In one cohort^31^, using both methods, MSI percentage was reported as 12.7% by IHC method and 8.2% by electrophoresis of DNA respectively (Table 1). As electrophoresis of DNA method was recommended by National Cancer Institute (NCI), we adopted 8.2% in our study and grouped this cohort in MSI ≤ 10% subgroup.

Immunohistochemistry (IHC) was the only method applied to detect the expression of ARID1A. Cutoff levels for ARID1A deficiency was defined as cancer cells weak or without nuclear staining, or nuclear staining <10%. All of the 14 studies with sample size from 66 to 489 reported OS, but only two studies reported the disease free survival (DFS) and one study reported the progression free survival (PFS). Thus, only OS was used as the endpoint of this study.

### Meta-analysis

**Figure 2:**
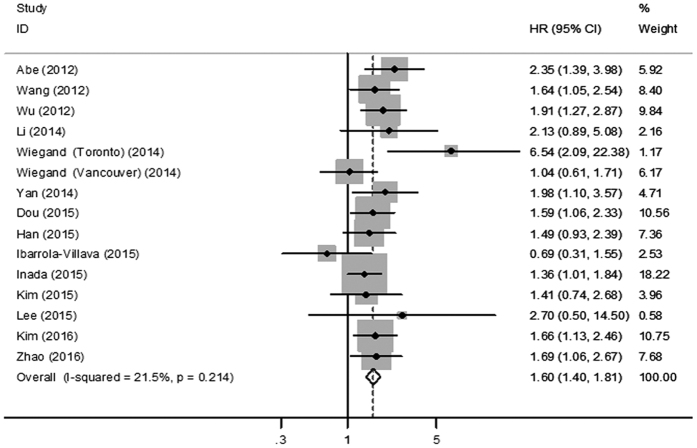
Forest plot of HR for ARID1A loss and overall survival. HR, hazard ratio; CI, confidence interval.

The results of meta-analyses for all studies are shown in Fig. 2. The loss of ARID1A expression was significantly associated with poor OS (HR = 1.60; 95% CI = 1.40–1.81; *P* < 0.001) in patients with GC (Fig. 2). The heterogeneity test showed low heterogeneity (*I*^*2*^ = 21.5%; *P* = 0.214) among these studies (Table 2), which suggested good consistency and the results from the 14 studies can be pooled together.

### Stratified analysis

Subgroup analyses were stratified according to ethnicity, disease location, Epstein-Barr virus (EBV) infection rate, TNM staging, tumor differentiation, status of MSI, ARID1A deficiency rate and sample size.

As listed in Table 2, the prognostic role of ARID1A expression loss was obvious in Asians (HR = 1.65, 95% CI = 1.44–1.89, *P* < 0.001), subgroup of disease in upper third part of stomach ≤30% (HR = 1.80, 95% CI = 1.36–2.38, *P* < 0.001) and subgroup of EBV (+) > 5% (HR = 1.59, 95% CI = 1.18–2.15, *P* = 0.002). In contrast, ARID1A expression loss did not significantly related to the poor outcome of GC patients in non-Asians (HR = 1.50; 95% CI = 0.53–4.20, *P* = 0.444), or high proportion of proximal stomach (Upper third part of stomach >30%, HR = 1.20, 95% CI = 0.40–3.61, *P* = 0.750) subgroup, or EBV (+) ≤ 5% subgroup (HR = 2.20, 95% CI = 0.93–5.22, *P* = 0.073). It seemed that ethnicity, disease location and EBV infection status might affect the correlation between ARID1A loss and poor OS. However, we found ARID1A expression loss was correlated with poor OS in GC, irrespective of tumor clinical stage, tumor differentiation, MSI status, ARID1A deficiency rate and sample size (Table 2).

### Sensitivity analysis and Publication bias

Sensitivity analysis was conducted by sequentially removing each study from pooled analysis to evaluate the robustness of the meta-analysis results. As shown in Table 3, the omission of single study didn’t statistically change the results of pooled HRs, indicating that our meta-analysis results were apparently stable and reliable.

**Figure 3:**
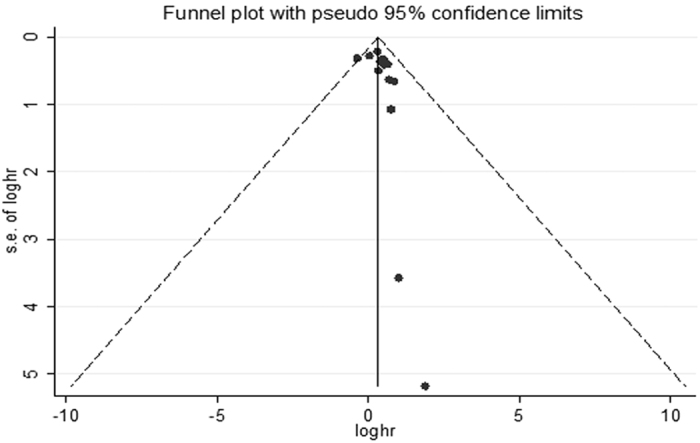
Funnel plot for included studies in the meta-analysis.

Begg’s funnel plot and Egger’s test were used to assess the publication bias. The shape of the Begg’s funnel plot seemed basically symmetrical by visual inspection (Fig. 3). Neither Begg’s test (*P* = 0.235) nor Egger’s test (*P* = 0.146) detected any evidence of publication bias.

## Discussion

To the best of our knowledge, this is the most comprehensive meta-analysis exploring the prognostic role of loss of ARID1A in GC patients. Our study revealed that loss of ARID1A expression was an indicator of poor prognosis in GC patients. The pooled hazard of death in ARID1A expression loss group was assessed to be 60% up compared with ARID1A expression group. Results from heterogeneity testing, sensitivity analysis and publication bias confirmed the reliability of our finding. The correlation between ARID1A expression loss and poor OS remained in subgroup analyses, regardless of tumor clinical stage, deficiency rate of *ARID1A* expression, sample size, tumor differentiation and MSI status. In addition, when stratification was analyzed by ethnicity, disease location and EBV status, ARID1A expression loss could predict poor OS for GC patients in Asians, proportion of proximal disease ≤30% and EBV (+) > 5% subgroup, but not in non-Asians, or proportion of proximal disease >30% or EBV (+) ≤ 5% subgroup.

In 2015, a meta-analysis published by Luchilin *et al*. investigated the prognostic role of mutation status of ARID1A in a variety of cancers and demonstrated that ARID1A expression loss was not connected with all-cause mortality in gastrointestinal cancer patients^25^, which is not consistent with our finding. The conflict findings call for the need of more in-depth investigation. However, we believe that our study yielded stronger evidence because our results were derived from more Chinese literatures, more GC patients from Eastern Asia, and a greater number of GC patients (3183 versus 1176 patients in the study by Luchilin *et al*.). Moreover, the results from heterogeneity test, sensitivity analyses and publication bias test showed that our findings are stable.

Since Huang *et al*. gave the evidence that ARID1A emerged as a tumor suppressor in 2007 36, the molecular mechanisms underlying worse outcome in cancer patients with ARID1A expression deficiency have been unveiled gradually^14^,^17^. It is thought that ARID1A contributes to tumor suppression in three main aspects. First, as a gatekeeper, ARID1A is capable of regulating cellular proliferation by directing cell cycle or promoting apoptosis. Study *in vitro* has revealed that silencing of *ARID1A* expression enhanced the proliferation and colony formation of GC cells, whereas restoring *ARID1A* expression led to the reverse effect^19^. Further functional study has exhibited that, ARID1A, collaborating with p53, regulated several downstream target genes, such as *CDKN1A* (p21) and *SMAD3*, to arrest cell cycle^17^. Second, *ARID1A* can functions as a “caretakers” by preventing genomic instability. Recent data has shown that *ARID1A* played a vital role in regulating DNA damage checkpoint and subsequently augmented DNA damage signaling^37^. Last, a growing body of evidence suggests that activation of some genes or pathways may act in concert with ARID1A loss in accelerating cancer development. For example, alteration in the PI3K/Akt pathway and TP53 status were found to be correlated with loss of ARID1A expression in ovarian clear carcinoma, endometrial cancer, and GC^18^,^19^,^38^,^39^.

In GC, Tumor stage and tumor differentiation have been manifested to be crucial clinical prognostic markers^5^,^40^. Whereas the prognostic role of MSI, which results from inactivation of DNA mismatch repair systems^41^, remains uncertain in GC^42^. It has been reported that loss of ARID1A expression was significantly correlated with tumor stage^22^,^31^, differentiation grade^32^ and MSI status^22^,^32^ in GC. For example, Wang revealed that loss of ARID1A expression was significantly linked to T stage and differentiation grade^22^. Kim *et al*. found that complete loss of ARID1A expression was 57.7% in poorly differentiated gastric adenocarcinomas, much higher than that in moderately differentiated GC (25%) and well differentiated GC (7.7%)^32^. Wang and Han respectively reported *ARID1A* gene frequently mutated in MSI-high GC^18^,^31^. However, it is ambiguous whether these factors mediate the association between loss of ARID1A expression and poor OS. Our subgroup analyses revealed that the prognostic role of ARID1A deficiency in GC was independent of tumor stage (T and N), differentiation grade and MSI status. In addition, neither deficiency rate of ARID1A expression nor sample size had effect on the relationship between ARID1A expression loss and prognosis in GC. These results indicated that ARID1A expression loss was a strong and stable prognostic biomarker for GC.

Notably, the subgroup analyses by ethnicity revealed that ARID1A expression loss had a significantly adverse impact on the OS in GC patients in Asians, but not in non-Asians. Similarly, this correlation between ARID1A loss and worse OS could be detected in proportion of proximal disease ≤30% subgroup, but not in high proportion of proximal disease subgroup. It seemed that ethnicity and disease location might be potential critical factors which influence the relationship between ARID1A loss and poor prognosis in GC. Tumor location varies markedly by ethnicity and geographic area. For example, non-proximal GC predominates in Japan and Korea, while proximal GC occurs more often in western countries^43^,^44^. Such variation in tumor location, in combination with genetic background may result in differences in tumor behavior and outcome^45^,^46^, which might consequently obscure the significance of *ARID1A* expression loss in prognosis.

EBV associated gastric carcinoma (EBVaGC), often diagnosed in non-antrum of stomach, happens more frequently in western countries^47^,^48^,^49^. Though some studies have demonstrated that lack of ARID1A loss expression is frequent in EBVaGC^31^,^34^, the prognostic role of ARID1A loss in EBVaGC remains unproven^50^,^51^. Our study showed that poor prognostic significance of ARID1A in GC existed in EBV (+) > 5% subgroup, which was not concordant with the findings that ARID1A expression loss could predict poor OS in Asians and proportion of proximal disease ≤30% subgroup. It is noteworthy that the individual-level data on EBV infection status were scanty, therefore the correlations between EBV infection status, tumor location and ethnicity were not further explored in our study.

As a literature-based meta-analysis, it is vital to note the limitations of our study. First, all of the included studies were derived from retrospective data, potentially leading to selection bias. Thus, prospective studies are requisite to confirm our finding in future. Second, only two studies, which consisted of 3 cohorts and represented only 10% of the total cases, were from non-Asian population. Thus, the conclusion in non-Asians was less persuasive. More original studies in non-Asian GC patients are necessary in future. Third, several HRs were extracted from survival curves, which might bring in small errors. However, our sensitivity analyses did not materially alter the results, which suggested that the effects due to such errors were limited. Finally, these included studies didn’t provide information about chemotherapy or radiotherapy, hence the issue whether chemotherapy and radiotherapy can influence the correlation between ARID1A expression loss and survival in GC was not investigated in our study.

Despite the above limitations, this is the most comprehensive meta-analysis, to date, to quantitatively assess the prognostic value of ARID1A expression loss in GC patients. Our results suggest that ARID1A inactivity is significantly related to poor OS for GC patients, which may be used to identify patients with poor outcome and guide clinical treatment modulation. Notably, our subgroup analyses further indicate that ethnicity and tumor location might be key influence factors for this connection, although the sample sizes of some subgroups are relatively small. In the future, larger scale prospective studies are warranted to confirm ARID1A expression loss as a prognostic role in GC patients. Moreover, since multi-markers may provide more precise prognostic information than single indicator, studies estimating ARID1A expression loss in coordination with other prognosis markers are essential to assess their value in GC survival.

## Methods

### Publication searching strategies

Literature search was undertaken, using Pubmed, Embase, Web of Science, Cochrane library, Chinese National Knowledge Infrastructure (CNKI) and Wanfang Database for both English and Chinese language articles. Last search was updated in March 3rd, 2016. The searching strategy contained the following terms with varied combination: GC (“gastric carcinoma” or “gastric neoplasm” or “GC” or “cancer of stomach” or “gastric tumor”), ARID1A (“ARID1A” or “BAF250a”) and prognosis (“prognosis”, “prognostic” or “outcome”). The reference lists of retrieved publications were further reviewed manually to identify potentially relevant articles.

### Study Inclusion and exclusion criteria

Studies included in this meta-analysis had to meet all of the following criteria: (1) patients recruited with histologically proven GC; (2) investigated the association between ARID1A deficiency and prognosis; (3) ARID1A deficiency was tested in primary gastric tumor tissue by immunohistochemistry (IHC), exome sequencing or reverse transcription-polymerase chain reaction; (4) the hazard ration (HR) and its 95% confidence interval (CI) were reported or could be calculated. In the case of duplicated publications or overlapped data, the most recent or more comprehensive article was included. In accordance with the inclusion criteria, two reviewers (L.Y. and H.H.X) performed the eligibility assessment independently, and the disagreements between the two reviewers were resolved by consensus.

### Data extraction

The following information was extracted from the included studies: first author, publication year, country of origin, sample size, clinical stage, location differentiation grade, ARID1A expression assay (methods, cutoff level and rate) and survival data. If univariate and multivariate HRs and their 95% CI were both reported, multivariate HRs were used.

### Extraction of hazard ratio

HRs and their 95% CIs were used to conduct the meta-analysis in this study. When they were given in literatures, we obtained them directly. When they were not described directly, we extracted them from available numerical data or published survival curves using methods reported by Parmar^52^ and Tierney^53^.

### Statistical analysis

An observed meta-HR > 1 implied a worse prognosis for the ARID1A deficiency group if its 95% CI didn’t overlap 1 (*P* < 0.05). The heterogeneity among the included studies was evaluated using the Cochran *Q*-test and *I*^*2*^ test. If a *P* ≤ 0.10 in Cochran Q test or *I*^*2*^ value ≥ 50% in *I*^*2*^ test, the heterogeneity was regarded as statistically significant. If there is no significant heterogeneity, fixed-effects models were used. Otherwise, random-effects models were performed. Subgroup analyses, according to ethnicity, clinical stage, differentiation grade, MSI and so on, were conducted to explore the factors which may influence the effect of ARID1A deficiency on prognosis. Sensitivity analysis was performed to examine the stability of the pooled results. A funnel plot with Begg’s and Egger’s test was applied to assess the publication bias and *P* > 0.05 was considered for no publication bias. All statistical analyses were performed using STATA Statistical Software, version 12.0 (Stata Corporation, College Station, TX).

## Additional Information

**How to cite this article**: Yang, L. *et al*. Loss of ARID1A expression predicts poor survival prognosis in gastric cancer: a systematic meta-analysis from 14 studies. *Sci. Rep.* **6**, 28919; doi: 10.1038/srep28919 (2016).

## Figures and Tables

**Table 1:** Characteristics of studies included in this meta-analysis.

Author	Year	Country		Sample size	Lauren differentiation (diffuse, %)	WHO differentiation (G3, %)	T1-2 (%)	N0 (%)	Location (U, %)	MSI	EBV (+) (ISH)	ARID1A (−) (%)	OS HR-E	HR (95% CI)
Abe	2012	Japan	250		53.3%	NA	NA	NA	27.9%	14.4% (I)	3.2%	11.0%	SC	2.35 (1.39, 3.98)
Wang	2012	China	224		NA	78.6%	26.4%	40.2%	NA	NA	NA	51.3%	HR	1.64 (1.05, 2.54)
Wu	2012	China	166		NA	65.7%	31.3%	18.7%	NA	NA	NA	24.1%	RR	1.91 (1.27, 2.87)
Yan	2014	China	176		NA	51.7%	30.1%	33.0%	NA	NA	NA	25.0%	HR	1.98 (1.10, 3.57)
Li	2014	China	90		NA	61.1%	15.6%	24.4%	50.0%	NA	NA	70.0%	SC	2.13 (0.89, 5.08)
Wiegand (Toronto)	2014	Canada	80		25.0%	57.5%	22.5%	40.0%	NA	21.3% (I)	2.5%	20.0%	HR	6.54 (2.09, 22.38)
Wiegand (Vancouver)	2014	Canada	173		21.4%	51.5%	10.4%	24.0%	NA	14.5% (I)	3.5%	22.5%	HR	1.04 (0.61, 1.71)
Dou	2015	China	103		37.9%	37.9%	42.7%	54.4%	NA	NA	NA	65.0%	RR	1.59 (1.06, 2.33 )
Han	2015	Korea	417		40.5%	36.9%	26.9%	NA	NA	8.2% (P)	7.3%	21.1%	SC	1.49 (0.93, 2.39)
Ibarrola- Villava	2015	Spain	66		36.4%	30.3%	NA	NA	30.3%	15.2% (P)	NA	27.0%	SC	0.69 (0.31, 1.55)
Inada	2015	Japan	489		NA	60.5%	NA	24.1%	NA	7.8% (I)	NA	39.6%	HR	1.36 (1.01, 1.84)
Kim	2015	Korea	191		37.7%	64.4%	50.3%	45.5%	13.1%	19.9% (I)	NA	32.5%	SC	1.41 (0.74, 2.68)
Lee	2015	Korea	275		46.9%	NA	56.7%	47.3%	15.3%	6.4% (P)	NA	8.0%	SC	2.70 (0.50, 14.50)
Kim	2016	Korea	350		32.3%	58.8%	33.7%	31.1%	20.0%	18.9% (I)	10%	18.6%	HR	1.66 (1.13, 2.46)
Zhao	2016	China	133		62.4%	70.7%	12.0%	18.8%	NA	NA	NA	42.1%	HR	1.69 (1.06, 2.67)

NA: Not available; U: Upper third part of stomach; MSI: Microsatellite instability; I: Immunohistochemistry of mismatch repair proteins; P: Polymerase chain reaction and electrophoresis of DNA; EBV: Epstein-Barr virus; ISH: *In situ* hybridization; OS: Overall Survival; HR-E: HR Estimated; SC: Survival curve; HR: Hazard ratio; RR: Relative risk; CI: Confidence interval.

**Table 2:** The loss of ARID1A expression and overall survival in GC.

**Outcome**	**Cohort** (**NO**.)	**Patient** (**NO**.)	**HR** (**95% CI**)	***P***	***I***^2^(**%**)	***P***_***H***_
Overall	15	3183	**1.60 (1.40, 1.81)**	**<0.001**	21.5%	0.214
Ethnicity						
Asian	12	2864	**1.65 (1.44, 1.89)**	**<0.001**	0.0%	0.915
Non-Asian	3	319	1.50 (0.53, 4.20)	0.444	79.9%	0.007
Location						
U ≤ 30%	4	1066	**1.80 (1.36, 2.38)**	**<0.001**	0.0%	0.589
U > 30%	2	156	1.20 (0.40, 3.61)	0.750	71.2%	0.062
EBV						
≤5%	3	503	2.20 (0.93, 5.22)	0.073	79.7%	0.007
>5%	2	767	**1.59 (1.18, 2.15)**	**0.002**	0.0%	0.725
T1,2						
≤30%	6	1117	**1.65 (1.21, 2.25)**	**0.002**	42.3%	0.123
>30%	6	1261	**1.72 (1.41, 2.10)**	**<0.001**	0.0%	0.935
N0						
≤30%	5	958	**1.81 (1.32, 2.50)**	**<0.001**	47.5%	0.107
>30%	7	1492	**1.55 (1.29, 1.88)**	**<0.001**	0.0%	0.721
Differentiation						
G3 ≤ 60%	7	1365	**1.52 (1.13, 2.06)**	**0.006**	52.5%	0.049
G3 > 60%	6	1293	**1.59 (1.32, 1.90)**	**<0.001**	0.0%	0.780
Lauren classification						
Diffuse ≤ 40%	6	963	**1.45 (1.01, 2.08)**	**0.042**	57.5%	0.038
Diffuse > 40%	4	1075	**1.79 (1.36, 2.36)**	**<0.001**	0.0%	0.594
MSI						
≤10%	3	1181	**1.42 (1.10, 1.82)**	**0.006**	0.0%	0.712
>10%	6	1110	**1.58 (1.03, 2.41)**	**0.036**	65.4%	0.013
ARID1A loss						
≤20%	4	955	**2.32 (1.45, 3.71)**	**<0.001**	41.7%	0.162
>20%	11	2228	**1.51 (1.31, 1.74)**	**<0.001**	0.0%	0.505
Sample size						
≤200	9	1178	**1.61 (1.24, 2.08)**	**<0.001**	43.5%	0.078
>200	6	2005	**1.59 (1.33, 1.90)**	**<0.001**	0.0%	0.596

U: Upper third part of stomach; EBV: Epstein-Barr virus; MSI: microsatellite instability; HR: hazard ratio; CI: confidence interval; *P*_*H*_: the P value of Cochran *Q*-test for heterogeneity.

**Table 3:** Sensitivity analysis of hazard ratio for ARID1A expression loss and overall survival in gastric cancer.

**Study Omitted**	**HR** (**95% CI**)	***P***	***I***^***2***^	***P***_***H:***_
Abe (2012)	1.56 (1.37, 1.78)	<0.001	16.8%	0.270
Wang (2012)	1.59 (1.39, 1.82)	<0.001	27.0%	0.164
Wu (2012)	1.57 (1.37, 1.79)	<0.001	23.6%	0.199
Li (2014)	1.59 (1.39, 1.81)	<0.001	25.3%	0.181
Wiegand (Toronto) (2014)	1.57 (1.38, 1.79)	<0.001	0.0%	0.500
Wiegand (Vancouver) (2014)	1.64 (1.44, 1.87)	<0.001	13.3%	0.307
Yan (2014)	1.58 (1.39, 1.80)	<0.001	24.8%	0.186
Dou (2015)	1.60 (1.40, 1.83)	<0.001	27.1%	0.164
Han (2015)	1.61 (1.41, 1.83)	<0.001	26.7%	0.167
Ibarrola-Villava (2015)	1.63 (1.43, 1.86)	<0.001	4.1%	0.406
Inada (2015)	1.65 (1.44, 1.91)	<0.001	21.2%	0.224
Kim (2015)	1.61 (1.41, 1.83)	<0.001	26.5%	0.170
Lee (2015)	1.59 (1.40, 1.81)	<0.001	25.5%	0.179
Kim (2016)	1.59 (1.39, 1.82)	<0.001	26.9%	0.166
Zhao (2016)	1.59 (1.39, 1.82)	<0.001	26.9%	0.166

HR: hazard ratio; CI: confidence interval; *P*_*H:*_: the P value of Cochran *Q*-test for heterogeneity.
